# GDF10 inhibits proliferation and epithelial-mesenchymal transition in triple‐negative breast cancer via upregulation of Smad7

**DOI:** 10.18632/aging.101983

**Published:** 2019-05-31

**Authors:** Tian Zhou, Lei Yu, Jianjun Huang, Xueke Zhao, Yanwen Li, Yaxin Hu, Yu Lei

**Affiliations:** 1 Department of Breast Surgery, Affiliated Hospital of Guizhou Medical University, Guiyang, Guizhou 550004, China; 2 Prenatal Diagnosis Center, Affiliated Hospital of Guizhou Medical University, Guiyang, Guizhou 550004, China; 3 Department of Infectious Diseases, Affiliated Hospital of Guizhou Medical University, Guiyang, Guizhou 550004, China; * Equal Contribution

**Keywords:** triple‐negative breast cancer, growth differentiation factor-10, epithelial-mesenchymal transition, invasion, apoptosis

## Abstract

Triple-negative breast cancer (TNBC) cannot be treated with current hormonal therapies and has a higher risk of relapse than other breast cancers. To identify potential therapeutic targets for TNBC, we conducted microRNA sequencing (RNA-Seq) in human TNBC specimens and tumor-matched controls. We found that growth differentiation factor-10 (GDF10), a member of the TGF-β superfamily, was downregulated in tumor samples. Further analysis of GDF10 expression in a larger set of clinical TNBC samples using qPCR confirmed its downregulation and association with parameters of disease severity. Using human-derived TNBC cell lines, we carried out GDF10 under- and overexpression experiments, which showed that GDF10 loss promoted cell proliferation and invasion. By contrast, overexpression of GDF10 inhibited proliferation, invasion, and epithelial mesenchymal transition (EMT) via upregulation of Smad7 and E-Cadherin, downregulation of p-Smad2 and N-Cadherin, and reduction of nuclear Smad4 expression. In addition, overexpression of GDF10 reduced tumor burden and induced apoptosis in a TNBC xenograft mouse model. These findings indicate that GDF10 acts as a tumor suppressor in mammary epithelial cells that limits proliferation and suppresses EMT. Efforts aimed at restoring GDF10 expression may thus bring a long-sought therapeutic alternative in the treatment of patients with TNBC.

## INTRODUCTION

Breast cancer is a heterogeneous disease, and a leading cause of death among women worldwide [1,2]. Based on molecular marker variants, breast cancers are classified into various subtypes [1]. Among those, triple‐negative breast cancer (TNBC) characterized by estrogen receptor (ER), progesterone receptor (PR), and human epidermal growth factor receptor 2 (HER2) expression deficiency is the most aggressive form [1,3,4]. Patients with TNBC generally have poorer prognosis due to increased risks of local recurrence and distant metastasis [5,6]. The challenge of treating TNBC resides in its heterogeneity, the lack of specific molecular targets, and development of resistance to systemic chemotherapy [7]. Therefore, novel effective therapies for TNBC are imminently needed.

RNA arrays, microRNA sequencing, and protein arrays helped to illuminate the molecular mechanisms of TNBC [8]. The resulting datasets offer possibilities for researching its molecular bases and testing new therapeutic strategies from multiple angles [8]. This is aided by the burgeoning field of bioinformatics, which allows to analyze biological data derived from high-throughput methods [9]. Among those, RNA-Seq is a highly efficient method for transcriptome sequencing and detection of gene expression [10]. Its advantages over other methods include high technical reproducibility, low background noise, and a large dynamic range [11].

We conducted RNA-Seq analysis to identify differentially expressed genes (DEGs) between clinical TNBC specimens and tumor-matched controls and found significant downregulation of growth differentiation factor-10 (GDF10), a member of the transforming growth factor-β (TGF-β) superfamily [12]. GDF10 plays an important role in cell proliferation and differentiation and is also known as BMP-3B due to its close relationship with bone morphogenetic protein-3 (BMP3), another member of the TGF-β superfamily [12]. TGF-β signals through three cell surface receptors, namely TGFBR1/2/3 [13,14]. Stimulation of TGFBR2 activates TGFBR1, which phosphorylates Smad2/3. These in turn combine with Smad4 and the complex enters the nucleus to regulate the transcription of target genes [15,16]. In addition, Smad7 is an inhibitor of TGF-β signaling, which prevents TGF-β-dependent formation of Smad2/Smad4 complexes [17,18]. Interestingly, GDF10 expression is induced by TGF-β-SMAD2/3 signaling after activation of TGFBR3, and its expression was shown to suppress survival, migration, invasion, and epithelial-mesenchymal transition (EMT) in oral squamous cell carcinoma cells [12]. By combining in vivo and in vitro analyses and bioinformatics tools, in the current study we elucidate critical mechanisms by which GDF10 downregulation contributes to TNBC progression. The present data suggest that GDF10 could be a novel target for diagnosis, prognosis, and drug research to improve outcomes for patients with TNBC.

## RESULTS

### Differential gene expression analysis in clinical TNBC specimens

**Figure 1:**
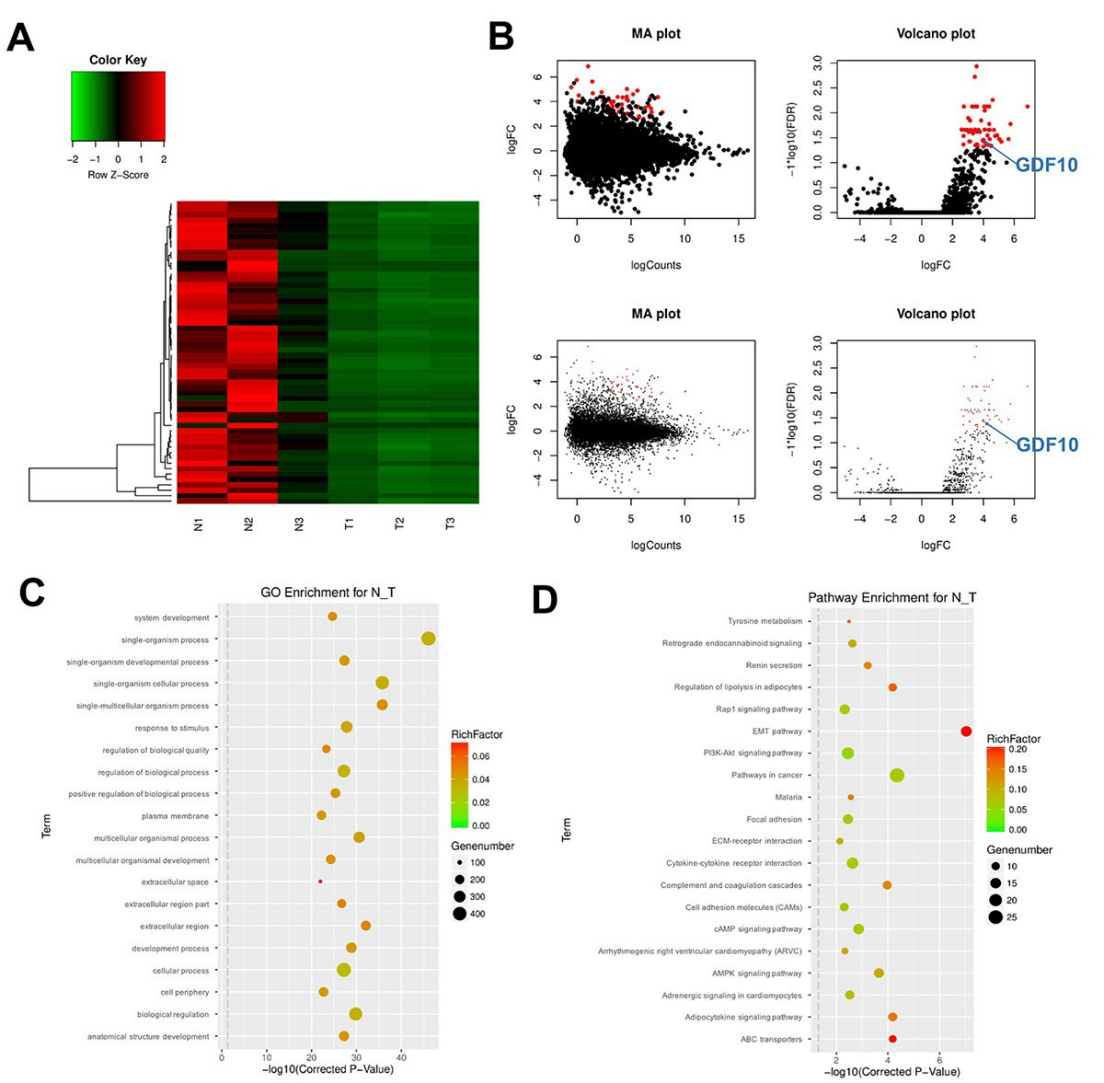
**DEG screening in clinical TNBC samples and matched control tissues.** (**A**) Heat Map showing the mRNA expression profiles of TNBC samples and adjacent normal tissues. Values correspond to the different colors representing the fold change (log2 transformed) of each sample. Black stands for 0 (no change in gene expression); red represents upregulation, and green represents downregulation. The distribution of genes with a change in expression of log2 fold-change (FC) ≥ 4 is represented in red in the volcano plot (log2 fold-change versus log FDR). (**B**) The distribution of genes with a false discovery rate (FDR) < 0.01 is marked in red on the MA plot (log total counts versus log2 fold-change, up panel). The distribution of genes with a change in expression of log2 fold-change (FC) ≥ 4 is represented in red on the volcano plot (log2 fold-change versus log FDR, up panel). (**C**) Gene ontology (GO) and (**D**) Kyoto Encyclopedia of Genes and Genomes (KEGG) analysis. Degree of enrichment is shown in the abscissa using corrected p values. Plot colors denote enrichment factor representing the ratio of DEG numbers to the total number of genes in the pathway. N1, N2, N3: normal tissues; T1, T2, T3: TNBC samples.

DEGs were analyzed in three clinical TNBC samples and their respective controls (adjacent normal tissue) using RNA-Seq and clustering analysis. As shown in Figure 1A, 56 downregulated mRNAs were detected in TNBC samples relative to controls. These DEGs were visualized in MA and Volcano plots with the criteria set as FDR < 0.01 or log2 fold-change (FC) ≥ 2 (Figure 1B).

Next, the DEGs were analyzed using Gene ontology (GO) enrichment and Kyoto Encyclopedia of Genes and Genomes (KEGG) pathway analyses. GO results indicated that DEGs were mainly enriched in the categories ‘response to stimulus’, ‘regulation of biological processes’, and ‘cellular processes’ (Figure 1C). Results of KEGG pathway analysis of DEGs indicated mainly the involvement of DEGs in the EMT pathway (Figure 1D). The enrichment analysis also showed that the EMT pathway was closely associated with cell growth and transport functions in TNBC.

### GDF10 expression is downregulated in TNBC specimens and cell lines

**Figure 2:**
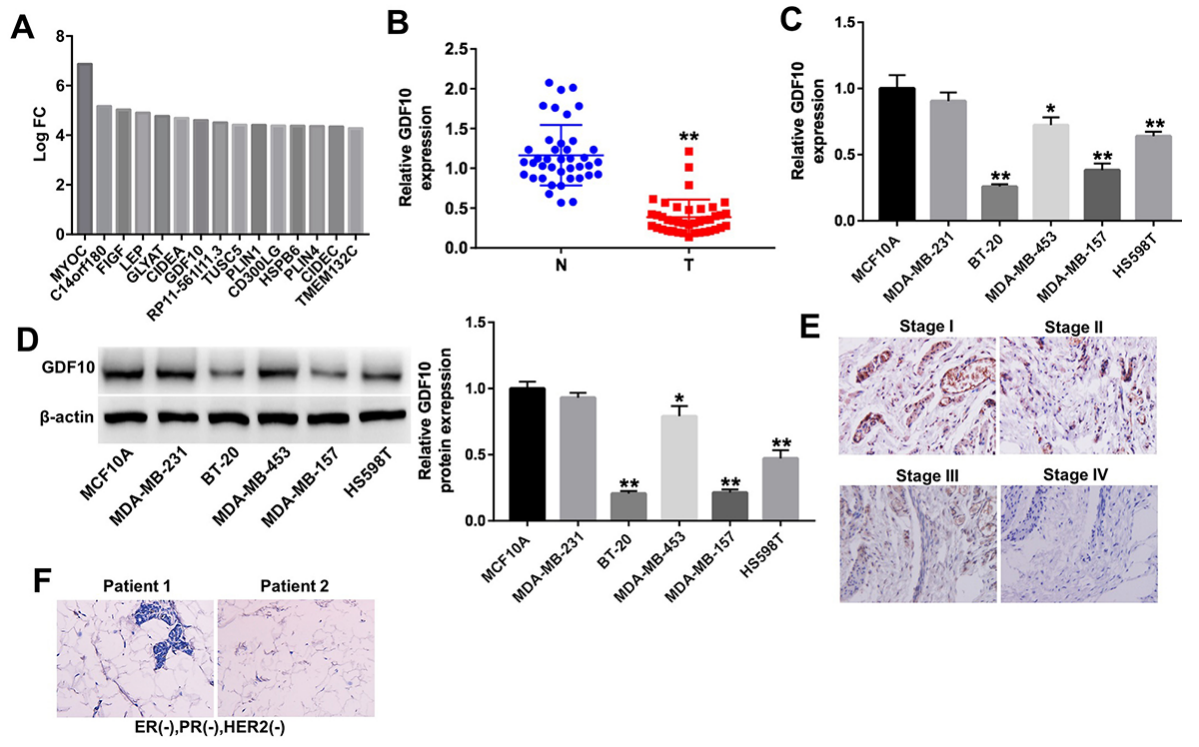
**GDF10 expression in human TNBC specimens and cell lines.** (**A**) Differentially expressed mRNAs (log2 fold-change (FC) ≥ 4) in TNBC samples. (**B**) Relative expression of GDF10 in tumors and adjacent normal tissues (N) from TNBC patients (n = 40). **P < 0.01, compared with the N group. (**C**) The levels of GDF10 in TNBC cell lines (MDA-MB-231, BT-20, MDA-MB-453, MDA-MB-157 and HS598T), and in normal mammary epithelial cells (MCF10A) were detected by qRT-PCR. *P < 0.05, **P < 0.01, compared with MCF10A cells. (**D**) GDF10 expression in MCF10A, MDA-MB-231, BT-20, MDA-MB-453, MDA-MB-157 and HS598T cells assessed by western blotting. β-actin was used as internal control. *P < 0.05, **P < 0.01, compared with MCF10A cells. (**E**) Representative GDF10 IHC staining (×200) of stages I, II, III, and IV TNBC samples. (**F**) IHC staining images of ER-negative nuclear expression, PR negative nuclear expression and HER-2/neu negative expression (magnification x 200) in patients with TNBC.

Among the DEGs identified in TNBC samples was GDF10, a secreted ligand of the TGF-beta superfamily of proteins previously associated with the EMT pathway [12], which showed significant downregulation (–log10(FDR) = 1.431; log2FC = 4.03; Fig. 2A). This finding was further corroborated in 40 additional clinical TNBC samples using qPCR. As shown in Figure 2B, the expression of GDF10 was significantly downregulated in tumor tissues compared with normal, matched controls. Moreover, GDF10 expression correlated with several clinicopathological parameters, including Ki67 expression and TNM stage (Table 1).

**Table 1:** GDF10 expression correlate with clinic-pathological parameters of patients with TNBC.

**Parameters**	**Number**	**GDF10**	***p* value**
**Age**			0.414
≤ 50	12	0.336 ± 0.220	
> 50	28	0.392 ± 0.201	
**Tumor volume**			
≤ 2 cm	18	0.496 ± 0.251	0.008**
> 2 cm	22	0.226 ± 0.122	
**Ki67**			0.023*
≤ 35%	16	0.447 ± 0.242	
> 35%	24	0.285 ± 0.102	
**Lymph node metastasis**			0.321
N0	11	0.368 ± 0.181	
N1-N3	29	0.315 ± 0.236	
**Distant metastasis**			0.046*
M0	22	0.421 ± 0.182	
M1	18	0.279 ± 0.208	
**TNM stage**			0.006**
I-II	19	0.426 ± 0.311	
III- IV	21	0.261 ± 0.209	

Student’s t test, *P<0.05, **P<0.01.

Next, qPCR and western blotting were used to detect the expression of GDF10 in five TNBC cell lines, MDA-MB-231, BT-20, MDA-MB-453, MDA-MB-157 and HS598T and in human breast epithelial MCF10A cells, used as non-tumorigenic control. In agreement with the findings described above, the mRNA and protein expression levels of GDF10 was significantly lower in BT-20, MDA-MB-157 and HS598T cells compared with MCF10A cells, respectively (Figures 2C and 2D). However, the level of GDF10 in MDA-MB-231 cells were not different compared with that in MCF10A cells, the difference might be the different types of TNBC cells (Figures 2C and 2D). In addition, densitometric analysis of IHC staining of human TNBC samples showed significantly decreased GDF10 expression in stage III/IV specimens, compared with stage I/II (Figure 2E). The represented IHC picture for ER, PR and HER2 staining was presented in Figure 2F. These results indicate that the expression of GDF10 is markedly downregulated in late-stage TNBC.

### Downregulation of GDF10 promotes proliferation of TNBC cells

**Figure 3:**
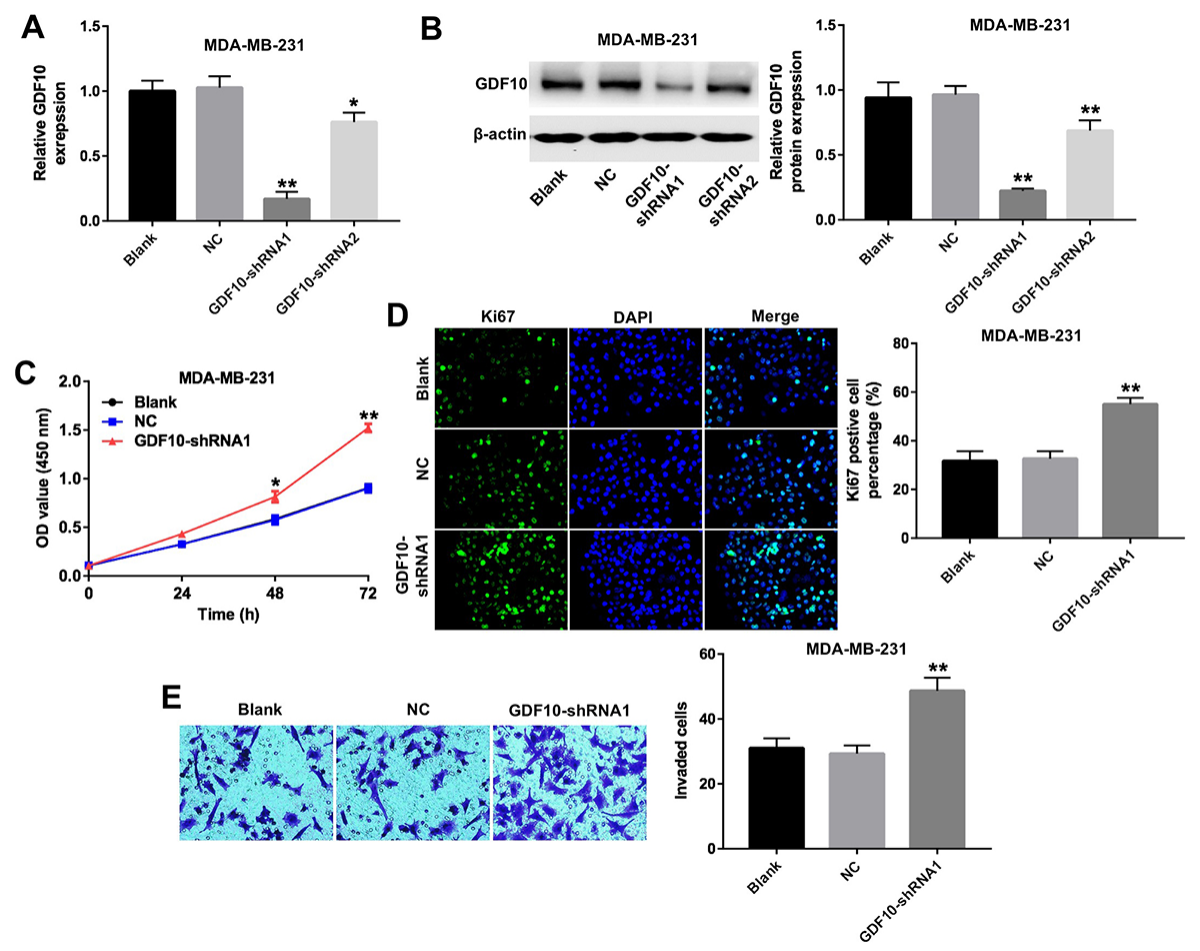
**Downregulation of GDF10 promotes proliferation of MDA-MB-231 cells.** GDF10 expression at the mRNA (**A**) and protein (**B**) levels after transfection with non-coding negative control shRNA (NC), GDF10-shRNA1, and GDF10-shRNA2. *P < 0.05, **P < 0.01, compared with the NC group. (**C**) Cell proliferation assay. MDA-MB-231 cells were transfected with NC, GDF10-shRNA1, and GDF10-shRNA2 and proliferation measured with the CCK-8 assay at 0, 24, 48, and 72 h. *P < 0.05, **P < 0.01, compared with the NC group. (**D**) Quantification of Ki67 expression by immunofluorescence in MDA-MB-231 cells. **P < 0.01, compared with the NC group. (**E**) Cell invasion assay. MDA-MB-231 cells were transfected with NC or GDF10-shRNA1 for 72 h and cell invasion assessed in Matrigel-coated transwell inserts. **P < 0.01, compared with the NC group.

To determine the function of GDF10 in TNBC, we used two different shRNAs (GDF10-shRNA1 and GDF10-shRNA2) to knock down its expression in the human TNBC cell line MDA-MB-231. Both qPCR and western blot results confirmed significant downregulation of GDF10 after transfection with GDF10-shRNAs (Figures 3A and 3B). Results showed cell viability was significantly increased in MDA-MB-231 cells following transfection with GDF10, compared to cells transfected with a non-targeting shRNA (NC group; Figure 3C). In addition, knockdown of GDF10 slightly increased proliferation in human breast epithelial MCF10A cells (Supplementary Figure 1A and 1B).

Furthermore, Ki67 expression is indicative of cells in a proliferative state [19]. n immunofluorescence assays, knockdown of GDF10 markedly increased the number of Ki67-postive MDA-MB-231 cells compared with NC controls (Fig. 3D).

Transwell invasion assays were performed to investigate the invasive capacity of MDA-MB-231 cells after transfection with GDF10-shRNA1. Results showed that knockdown of GDF10 markedly increased cell invasion (Figure 3E).

### Overexpression of GDF10 inhibits proliferation of TNBC cells

**Figure 4:**
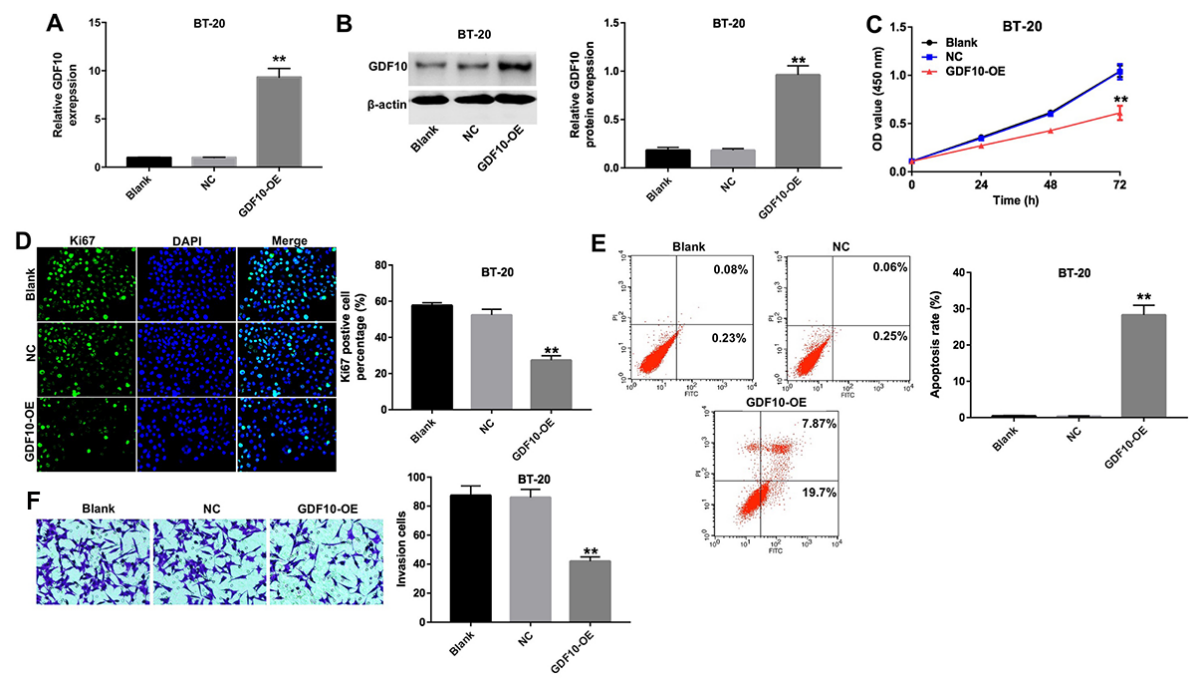
**Overexpression of GDF10 inhibits proliferation of BT-20 cells.** GDF10 expression at the mRNA (**A**) and protein (**B**) levels in BT-20 cells 72 h after transfection with NC or GDF10 for 72 h. **P < 0.01, compared with the NC group. (**C**) Cell proliferation assay results. BT-20 were transfected with NC or GDF10 and the CCK-8 assay was conducted at 0, 24, 48, and 72 h. **P < 0.01, compared with the NC group. (**D**) Ki67 immunofluorescence quantification in NC and GDF10-overexpressing BT-20 cells. **P < 0.01, compared with the NC group. (**E**) Apoptosis rates detected through Annexin V/PI double staining and flow cytometry in NC and GDF10-overexpressing BT-20 cells. **P < 0.01, compared with the NC group. (**F**) Cell invasion assay. BT-20 cells were transfected with NC or GDF10 for 72 h and Matrigel-coated transwell inserts used to quantify their invasive capacity. **P < 0.01, compared with the NC group.

To further confirm the impact of GDF10 on the proliferation of TNBC cells, we tested the effect of GDF10 overexpression on BT-20 cells (Figures 4A and 4B). Overexpression of GDF10 not only decreased proliferation (Figure 4C and 4D), but induced also apoptosis in BT-20 cells (Figure 4E). Nevertheless, overexpression of GDF10 neither inhibited proliferation, nor induced apoptosis in MCF10A (Supplementary Figure 1C, 1D, 1E and 1F). Additionally, upregulation of GDF10 markedly decreased the invasion of BT-20 cells in transwell assays (Figure 4G). These results indicate that GDF10 functions as a negative regulator of proliferation and invasion in TNBC cells.

### Overexpression of GDF10 induces cell cycle arrest and inhibits EMT in TNBC cells

**Figure 5:**
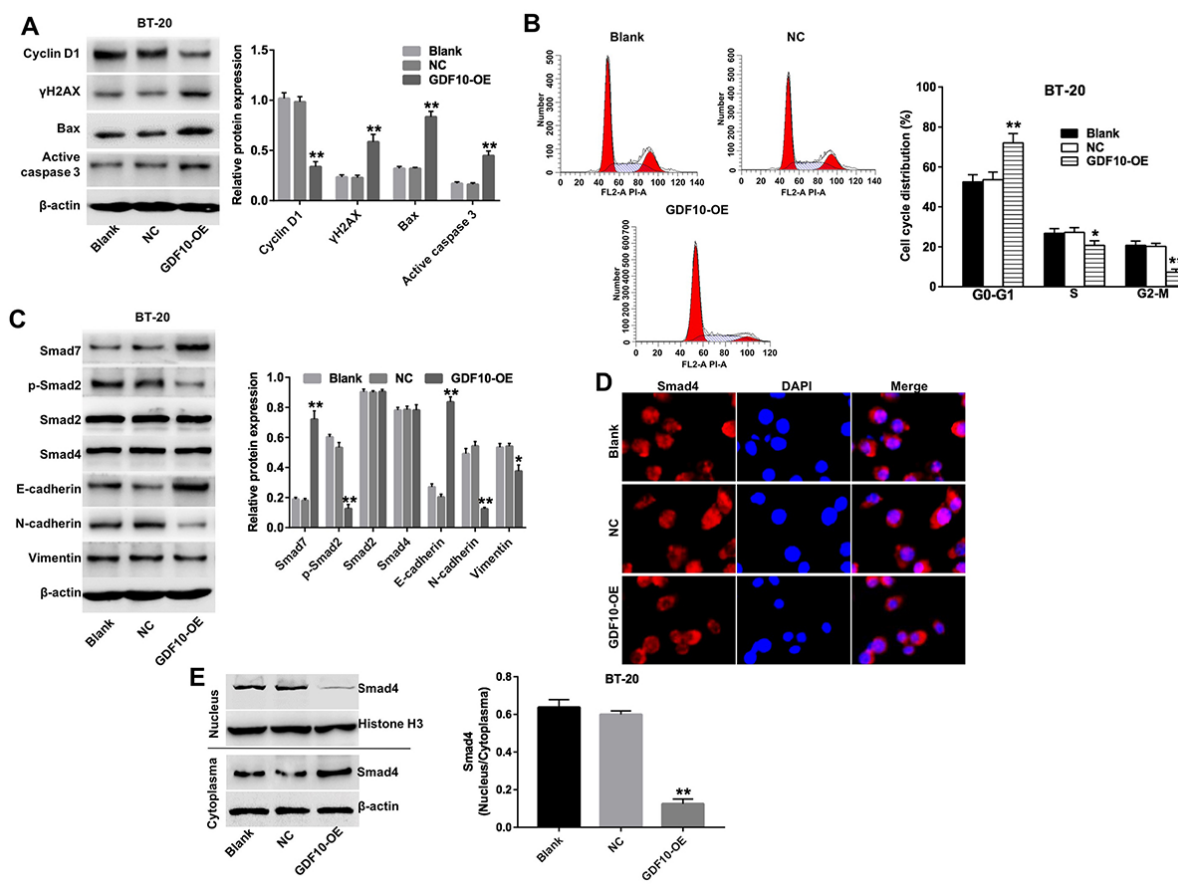
**Overexpression of GDF10 induces cell cycle arrest and inhibits EMT in BT-20 cells.** (**A**) The expression of cyclin D1, γH2AX, Bax, and active caspase-3 was investigated via western blotting in BT-20 cells transfected with GDF10 for 72 h. β-actin was used as internal control. Relative expression data were quantified by densitometry and normalized to β-actin. **P < 0.01, compared with the NC group. (**B**) Cell cycle distribution in BT-20 cells transfected with GDF10 for 72 h. *P < 0.05, **P < 0.01, compared with the NC group. (**C**) Smad7, p-Smad2, Smad2, Smad4, E-cadherin, N-cadherin, and Vimentin expression was assessed by western blotting in BT-20 cells transfected with GDF10 for 72 h. β-actin was used as internal control. Relative protein expression levels were quantified by densitometry and normalized to β-actin. *P < 0.05, **P < 0.01, compared with the NC group. (**D**) Immunofluorescent assessment of the subcellular distribution of Smad4 in BT-20 cells transfected with GDF10. (**E**) Smad4 expression was assessed by western blotting in the nucleus and cytoplasma of BT-20 cells transfected with GDF10 for 72 h, respectively. Histone H3 and β-actin were used as internal control respectively. Magnification × 400.

The effect of GDF10 on cell cycle progression was analyzed through western blotting and flow cytometry. Overexpression of GDF10 decreased the expression of cyclin D1 and active caspase 3, and increased the expression of γH2AX and Bax (Figure 5A). In addition, compared to NC cells the percentage of BT-20 cells in the resting phase (G0-G1) was increased, with a concomitant reduction in the number of cells in the proliferative phase (S) and the division phase (G2/M) (Figure 5B). These findings demonstrated that GDF10 mediates antiproliferative effects through induction of cell cycle arrest in TNBC cells.

Based on the inhibitory effects of GDF10 on cell invasion described above, we asked whether GDF10 would affect the expression of EMT related proteins in BT-20 cells. In western blot experiments, we found that overexpression of GDF10 downregulated the expression of p-Smad2, N-cadherin, and Vimentin, and upregulated the expression of Smad7 and E-cadherin (Figure 5C). There was no difference as for the expressions of Smad2 and Smad4 in each group (Figure 5C). In addition, the intracellular distribution of Smad4 was examined through immunofluorescence assays, revealing that nuclear accumulation of Smad4 was decreased after overexpressing GDF10 in BT-20 cells (Figure 5D). Similarly, western blot analysis revealed that upregulation of GDF10 markedly decreased the nuclear accumulation of Smad4 (Figure 5E). In summary, these observations suggested that GDF10 functions as a tumor suppressor in mammary epithelial cells by promoting cell cycle arrest and inhibiting EMT.

### GDF10 inhibits TNBC growth *in vivo*

**Figure 6:**
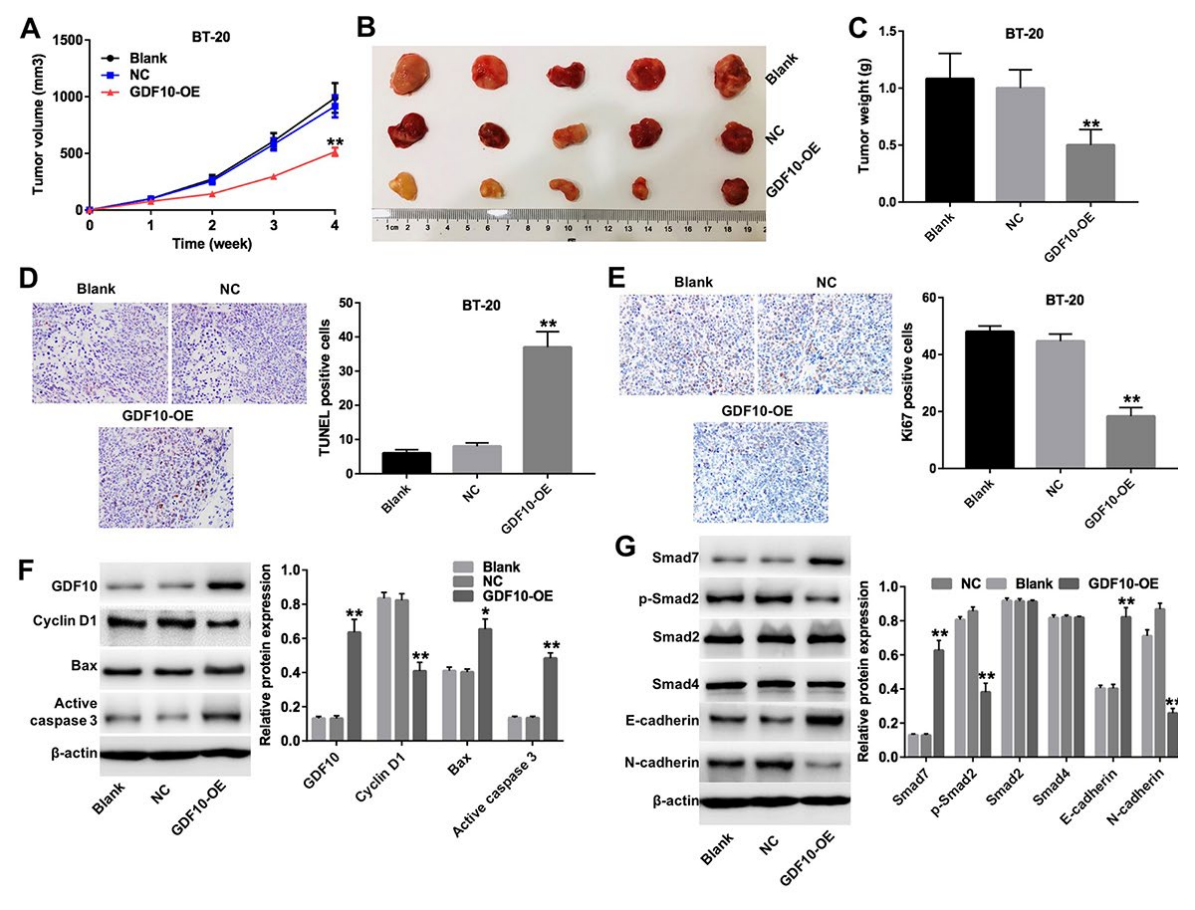
**GDF10 expression inhibits BT-20 xenograft growth.** (**A**) Tumor volumes were measured weekly post-inoculation of BT-20 cells infected with lentiviruses carrying the GDF10 gene or non-coding controls (NC). **P < 0.01, compared with the NC group. (**B**) Photographs of BT-20 xenografts dissected 4 weeks after tumor cell inoculation. (**C**) Tumor weights. **P < 0.01, compared with the NC group. (**D**) TUNEL staining of BT-20 tumors and quantification of TUNEL-positive cells. **P < 0.01, compared with the NC group. (**E**) Ki67 IHC in excised BT-20 tumor sections and quantification of Ki67-positive cells. **P < 0.01, compared with the NC group. (**F**) GDF10, cyclin D1, Bax, and active caspase-3 expression was assessed in tumor samples by western blotting. β-actin was used as internal control. Relative protein expression levels were quantified by densitometry and normalized to β-actin. *P < 0.05, **P < 0.01, compared with the NC group. (**G**) The expression of Smad7, p-Smad2, Smad2, Smad4, E-cadherin, and N-cadherin was investigated by western blotting in excised tumor samples. β-actin was used as internal control. Relative protein expression levels were quantified by densitometry and normalized to β-actin. *P < 0.05, **P < 0.01, compared with the NC group.

To further assess the antitumorigenic effects of GDF10 *in vivo*, BT-20 cells were infected with a lentivirus carrying the human GDF10 gene or a non-coding sequence (NC), and injected subcutaneously into female nude mice. Tumor xenografts derived from GDF10-overexpressing cells (GDF10-OE group) were significantly smaller (Figures 6A and 6B), and lighter (Figure 6C) than those derived from NC cells. Moreover, TUNEL staining indicated that overexpression of GDF10 resulted in prominent apoptosis in GDF10-overexpressing tumors, compared with the NC group (Figure 6D). In parallel, IHC staining showed reduced Ki67 expression in tumor sections from the GDF10-OE group (Figure 6E).

Next, we conducted western blot experiments that showed that the expression of GDF10, Bax, active caspase 3, Smad7, and E-cadherin was increased, while that of cyclin D1, p-Smad2, and N-cadherin was decreased, in the GDF10-OE group compared to the NC group (Figures 6F and 6G). In addition, the antitumorigenic effects of GDF 10 was confirmed by testing the other TNBC xenograft model (Supplementary Figure 2A, 2B, 2C and 2D). Thus, our *in vivo* data are consistent with our *in vitro* findings and further support the conclusion that GDF10 serves as a tumor suppressor that is downregulated in TNBC.

## DISCUSSION

The aim of the present study was to identify differentially expressed genes of clinical relevance in TNBC, usually the most aggressive form among breast cancer subtypes. Among the DEGs detected by RNA-seq in clinical TNBC specimens we focused on GDF10, a secreted TGF-β receptor ligand with growth factor activity whose downregulation was shown to contribute to oral carcinogenesis [12]. GO and KEGG analyses suggested the involvement of GDF10 in several cell processes, including the EMT pathway and revealed multiple, potentially relevant contributions to TNBC for other DEGs as well.

Using by qPCR and western blotting we confirmed significant downregulation of GDF10 in another 40 human TNBC samples and in human TNBC cell lines compared, respectively, to matched control tissues and normal mammary epithelial cells. Importantly, GDF10 expression in clinical samples correlated negatively with tumor proliferation (Ki67 staining) and TNM stage. Accordingly, IHC staining showed that GDF10 expression was markedly decreased in late TNBC stages, compared to early stages.

By implementing under- and over-expression experiments in vitro we showed for the first time that GDF10 acts as a tumor suppressor in TNBC by inducing cell cycle arrest, and inhibiting proliferation and invasion of immortalized cells. Based on the current data, and previous findings by Chen et al. in oral squamous cell carcinoma [12], and both Tandon et al. [20] and Dai et al. [21] in lung cancer, we propose a common role for GDF10 as a suppressor of epithelial carcinogenesis. Specific to breast cancer, Slattery et al. found that genetic variations in GDF10 were associated with ER-PR+ and ER-PR- breast cancer subtypes in a comparative study of hispanic and non-hispanic white women [22].

The mechanistic bases of the tumor-suppressing actions of GDF10 were investigated by Upadhyay et al., who found that Sca-1 disrupts GDF10-mediated TGFB signaling and promotes mammary tumorigenesis, while upregulation of GDF10 reverses this effect [23]. Our study further suggests that overexpression of GDF10 in TNBC cells can activate DNA damage-induced apoptosis by increasing the expressions of γH2AX, Bax, and active caspase 3. Bax, and active caspase 3 are the pro-apoptosis proteins, and γH2AX is a DNA double-strand break marker [24,25] Zhao et al indicated that berberine inhibits apoptosis in TNBC cells via increasing the expressions of γH2AX, Bax, and active caspase 3 [26]. In addition, overexpression of GDF10 obviously inhibited cell cycle progression via inducing G0/G1 phase cell cycle arrest. This finding was further supported by the decrease of cyclin D1, a cell cycle regulator [27]. When cells underlie DNA damage, the level of cyclin D1 was inhibited and then trigger G0/G1 phase arrest [28]. These observations are in line with evidence that a related protein, i.e. BMP-9, induces apoptosis and may act also as a tumor suppressor in prostate cancer cells [29].

**Figure 7:**
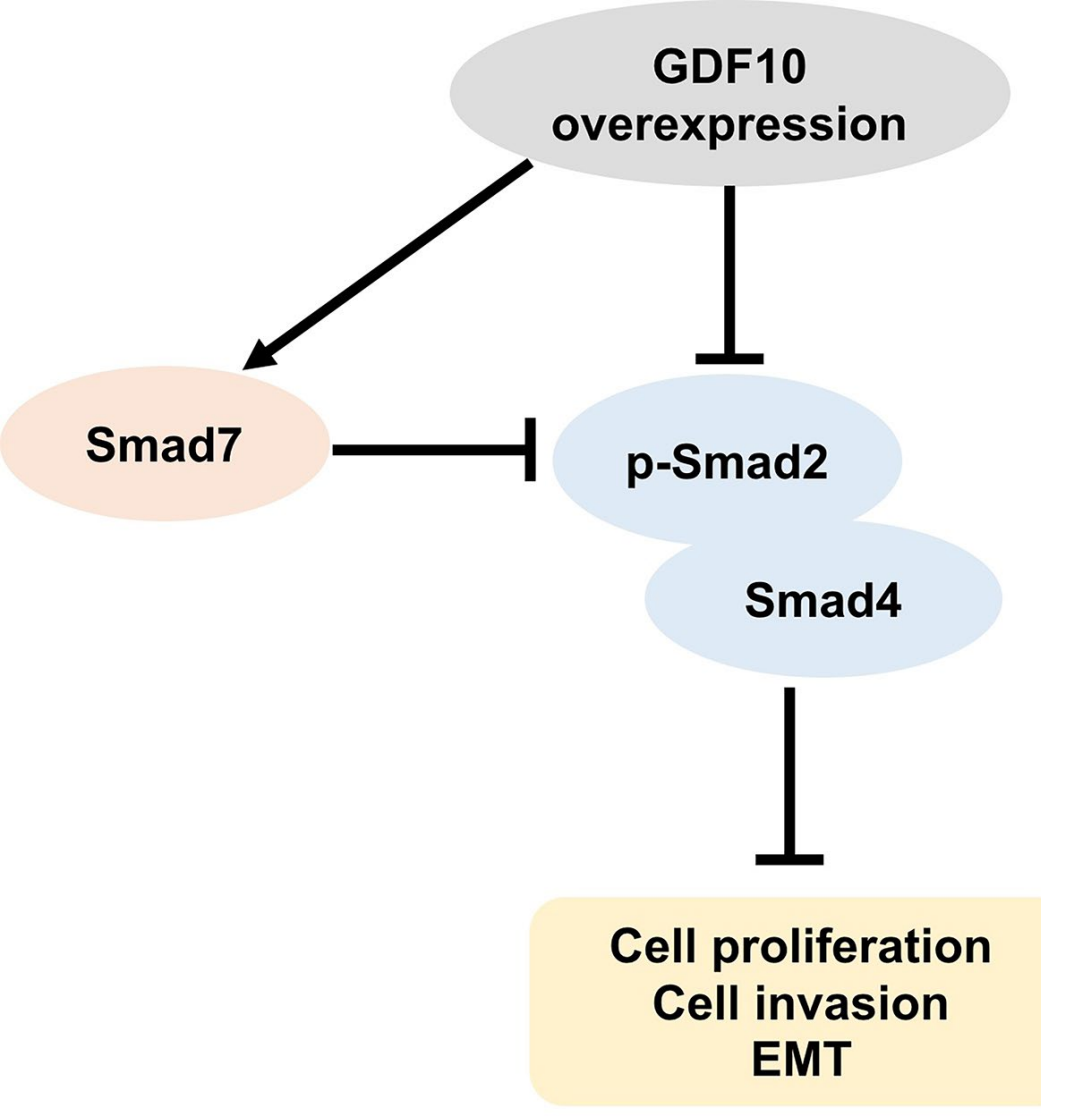
**Schematic model of the antitumor actions of GDF10 on TNBC cells.** GDF10 expression increases Smad7 levels and inhibits the formation of Smad2/4 complexes. Overexpression of GDF10 suppresses proliferation, EMT, and invasion in TNBC cells via inhibiting the formation of Smad2/4 involving the upregulation of Smad7.

Interestingly, we found that changes in GDF10 expression correlated with changes in the expression and subcellular localization of Smad proteins both *in vitro* and *in vivo*. In addition, TGF-β1/Smad pathway induced EMT via activating EMT transcription factor Snail gene [30]. Moreover, EMT transcription factor ZEB1 could bind phosphorylated Smad2/3 to enhance TGF-β1 [31]. EMT is associated with tumor recurrence and metastasis, all EMT subpopulations presented similar tumor-propagating cell capacity [32,33]. The transcription factors and signaling pathways could control these different EMT transition states [34]. This suggests a direct link between GDF10-mediated TGF-β signaling, EMT, and the metastatic capacity of TNBC cells and supports the idea that GDF10 inhibits cancer growth at least in part by inhibiting EMT. TGF-β1 negatively regulates EMT by various signaling processes, including a Smad4-dependent pathway, to mediate antitumor effects [35]. However, Smad7 can inhibit BMP/TGF-βs signaling in multiple ways [36] [37]. In addition, Smad7 blocks Smads signaling by inhibiting the phosphorylation of Smad2/3 [38]. Once Smad7 is degraded via the ubiquitin proteasome degradation mechanism, Smad2/3 is activated [39]. Our in vitro and in vivo experiments showed that overexpression of GDF10 increased Smad7 expression and inhibited the formation of Smad2/4 complexes, leading to reduced nuclear accumulation of Smad4. Hence, we propose that this mechanism is responsible for GDF10-mediated EMT inhibition in TNBC cells (Figure 7).

In summary, the current study showed that GDF10 is downregulated in both TNBC patient samples and immortalized cell lines, and its loss correlates with tumor aggressiveness. In contrast, GDF10 overexpression in TNBC cells inhibited proliferation and invasion by inducing G0 arrest and preventing EMT, while markedly reducing tumorigenicity and inducing apoptosis in a mouse xenograft model. Meanwhile, whether EMT transcription factors are involved in the regulation of GDF10 need to be investigated in the future studies.

Overall, our studies suggest that GDF10 acts as a tumor suppressor in mammary and other epithelial cells. Since targeted therapies for TNBC are still lacking, restoring GDF10 expression arises as an exciting and novel potential intervention to treat TNBC.

## MATERIALS AND METHODS

### Clinical samples

Forty pairs of matched TNBC and adjacent normal tissue samples were obtained in the Affiliated Hospital of Guizhou Medical University (Guizhou, China) from August 2011 to May 2017. Adjacent normal tissues 5 cm from tumor were collected during surgery. All samples were collected during surgical therapy and quick-frozen in liquid nitrogen. Informed written consent was obtained from all patients (age range: 25 to 55 years). The study was approved by the Ethics Committee of the Affiliated Hospital of Guizhou Medical University. Clinicopathological characteristics of patients are listed in Table 1.

### RNA sequencing

Total RNA from 3 TNBC samples (T1/2/3 group) and 3 matched normal tissues (N1/2/3 group) was extracted and purified using TRIzol reagent according to the manufacturer’s instruction (Invitrogen, Carlsbad, USA). The purity and quantity of initial total RNA samples were determined after DNase I treatment (New England Biolabs, Beijing, China) with a Nanodrop 2000 spectrophotometer (Thermo Fisher Scientific, DE, USA). An Agilent 2100 bio-analyzer (Agilent, CA, USA) was used for quality inspection to exclude genomic DNA (gDNA) contamination. RNA-seq was performed as reported before [40].

### GO and KEGG pathway analyses

Gene ontology (GO) enrichment is a widely used resource to find gene information. To analyze DEGs at the functional level, the hypergeometric test was executed to find biological functions enriched with such DEGs (http://mathworld.wolfram.com). P-values obtained from the hypergeometric test were corrected by the Benjamini-Hochberg method. DEG associations within Biological Processes (BPs), Cellular Components (CCs), and Molecular Functions (MFs) in the GO database were analyzed using the online tool DAVID (https://david.ncifcrf.gov). The criterion used in GO analyses was p ≤ 0.05. The Kyoto Encyclopedia of Genes and Genomes (KEGG) pathway analyses is helpful to identify biological pathways significantly altered under specific experimental conditions. KEGG enrichment analysis of DEGs was performed using the online tool KOBAS with p ≤ 0.05.

### Cell culture

Human breast epithelial (MCF10A) and TNBC (MDA-MB-231, BT-20, MDA-MB-453, MDA-MB-157 and HS598T) cell lines were purchased from American Type Culture Collection (ATCC, Rockville, MD, USA) and cultured in Dulbecco’s modified Eagle’s medium (DMEM, Gibco, USA) supplemented with 10% fetal bovine serum (FBS, Gibco), penicillin and streptomycin (100 U/ml, Thermo Fisher Scientific, Waltham, MA, USA) in a humidified incubator with 5% CO_2_ at 37˚C.

### Quantitative PCR

Total RNA from TNBC tissues, matched controls, and cells was extracted using the RNAsimple Total RNA kit (Tiangen Biotech Co., Ltd., Beijing, China) according to the manufacturer's instructions. cDNA was synthesized via reverse transcription using an oligo(dT) primer (Invitrogen, Carlsbad, CA, USA). Real-time PCR consisted of initial denaturation at 94˚C for 5 min and 40 thermal cycles of 94˚C for 45 s, 55˚C for 30 s, and 72˚C for 45 s. We used the SYBR premix Ex Taq II kit (TaKaRa, Dalian, China) on an ABI 7900HT instrument (ABI, NY, USA). Primers were purchased from GenePharma (Shanghai, China). GDF10: F: CCTACTACTGTGCTGGAGCC; R: TCTGGATGGTGGCATGGTTG. GAPDH: F: ATGGCCTTCCGTGTTCCTAC; R: CTTTACAAAGTTGTCGTTGA. All samples were run in triplicate. Relative quantification of gene expression was performed using the 2-ΔΔCT method to calculate fold-changes.

### Short hairpin RNA plasmids

Lentiviral vectors expressing short-hairpin RNA directed against human GDF10 (GDF10-shRNA1: 5’-GCGCCCUACAUCCUAGUCUAU-3’; GDF10-shRNA2: CCAATTGGATCTAACTCCATCCTCA) or a non-targeting RNA sequence (negative control) were purchased from GenePharma (Shanghai, China). Each plasmid vector expressed a shRNA under the control of a CMV promoter and contained a green fluorescent protein (GFP) reporter gene. Lentiviral DNA vectors were then transfected into 293T cells, followed by incubation at 32˚C to enhance viral titer. After 48 h, the supernatant containing the retroviral particles was collected, filtered through a low protein-binding syringe filter (0.45 μm) and the titer of lentiviruses was determined.

### GDF10 shRNA knockdown

MDA-MB-231 cells were plated onto 60 mm plates at 4 x 10^5^ cells/well. After 24 h, two GDF10-shRNAs supernatants were added directly to each cell culture (at 50-60% confluence). The virus-containing medium was replaced with fresh complete medium 24 h later. Stably transfected MDA-MB-231 cells were then selected by puromycin (2.5 μg/mL, Sigma Aldrich, St. Louis, MO, USA) over 3 days. Western blotting and qPCR assays were used to verify cellular expression of GDF10.

### Exogenous GDF10 overexpression

BT-20 cells were plated onto 60 mm plates at 4x10^5^ cells/well overnight. Then, supernatants with lentiviruses carrying the human GDF10 gene (GDF10-OE) were added directly to BT-20 cells (at 50-60% of confluence) for 24 h. Next, cells were re-plated on selection medium containing puromycin (2.5 μg/mL) for another 3 days. Western blotting and qPCR assays were used to verify GDF10 expression in the cells.

### Western blotting

MCF10A, MDA-MB-231, BT-20, and MDA-MB-453 cells were cultured in complete DMEM and collected in cell lysis buffer. The Bradford Protein Assay Kit (Beyotime, Shanghai, China) was used to measure protein concentration. Proteins were separated using 10% SDS polyacrylamide gels and transferred onto polyvinylidene fluoride (PVDF) membranes (Thermo Fisher Scientific) for 2 h. Membranes were blocked with 5% defatted milk in TBST for 1 h at room temperature, washed in TBST three times, and incubated with primary antibodies (purchased from Abcam): anti-GDF10 (ab325005; 1:1000), anti-Cyclin D1 (ab134175; 1:1000), anti-Bax (ab32503; 1:1000), anti-active caspase 3 (ab2302; 1:1000), anti-β-actin (ab8227; 1:1000), anti-γH2AX (ab2893; 1:1000), anti-Smad7 (ab216428; 1:1000), anti-Smad2 (ab40855; 1:1000), anti-p-Smad2 (ab184557; 1:1000), anti-E-cadherin (ab1416; 1:1000), anti-N-cadherin (ab18203; 1:1000), anti-Vimentin (ab8978; 1:1000), or anti-Histone H3 (ab8580; 1:1000). After washing, the PVDF membrane was incubated with an HRP-conjugated anti-rabbit secondary antibody (Abcam; ab7090; 1:5000). Signals were detected by chemiluminescence after incubation with ECL reagent (Santa Cruz Biotechnology, Dallas, TX, USA). Protein blot densities were normalized to β-actin.

### Cell proliferation assay

Proliferation was measured using the Cell Counting Kit-8 assay (CCK8, Beyotime, Shanghai, China). Cells (MDA-MB-231 and BT-20, 5,000 cells per well) were cultured in 96-well plates and transfected with GDF10-shRNA1 or GDF10-OE (GDF10 overexpression). Proliferation was measured at 0, 24, 48, and 72 h in triplicate. To this end, 10 μL CCK-8 reagent was added to each well for 2 h at 37°C. Absorbance was measured at 450 nm using a Thermo Multiskan FC microplate reader (Thermo Fisher Scientific).

### Immunohistochemistry

Progesterone Receptor (PR), estrogen receptor (ER), HER2, GDF10 and Ki67 expression was determined by immunohistochemical (IHC) staining according to methods reported before [41]. Briefly, the specimens were cut into 5-μm sections, placed on slides, and baked at 65°C for 2 h. The slices were incubated with the primary antibodies overnight, and biotinylated goat anti-rabbit IgG was applied for 30 min at room temperature. Visualization was performed using a polymer IHC detection system (EnVision kit; Dako Japan).

### Immunofluorescence

MDA-MB-231 and BT-20 cells were seeded onto 24-well plates overnight and transfected for 72 h with GDF10-shRNA1 or GDF10-OE. Cells were then washed in PBS three times, prefixed in 4% paraformaldehyde for 10 min at room temperature, and fixed in cold methanol for 10 min at -20°C. Next, cells were incubated with anti-Ki67 (ab15580; 1:1000) or anti-Smad4 (ab40759; 1:1000) primary antibodies (both from Abcam) at 4°C overnight. Subsequently, cells were incubated with secondary antibodies (Abcam; ab150080; 1:5000) at 37°C for 1 h and counterstained with DAPI. The samples were observed on a fluorescence microscope (Olympus CX23, Tokyo, Japan).

### Matrigel invasion assay

Cell invasion was assayed using 24-well transwell chambers (Corning, New York, NY, USA) according to the manufacturer's protocol. Briefly, the upper chamber was pre-treated with 100 μl of Matrigel and exposed to UV light for 2 h. MDA-MB-231 or BT-20 cells (1×10^5^) were seeded onto the upper chamber in serum-free medium, and the bottom wells filled with DMEM containing 10% FBS. After 72 h incubation at 37°C, cells on the upper surface of the filter were removed with a cotton swab. The cells on the underside of the membrane were fixed in 100% methanol and stained with a solution containing 50% isopropanol, 1% formic acid, and 0.5% crystal violet. Invading cells were counted in 3 randomly selected fields.

### Apoptosis assay

After transfection with GDF10 shRNA1 or GDF10-OE for 72 h MDA-MB-231 and BT-20 cells were washed with cold PBS and centrifuged at 1000 rpm/min for 5 min. The cell pellet was resuspended in 100 μL binding buffer and 5 μL Annexin V-FITC plus 5 μL propidium iodide (PI) were added. After incubating 15 min at room temperature, 200 μL binding buffer was added and apoptosis was evaluated by flow cytometry (BD, Franklin Lake, NJ, USA). The software WinMDI 2.9 (Invitrogen, CA, USA) was used to analyze results.

### Cell cycle analysis

Cell cycle distribution was analyzed by flow cytometry in BT-20 cells transfected with GDF10-OE for 72 h. Cells were washed in cold PBS once and fixed in cold 70% ethanol at 4°C overnight. Cells were then washed in cold PBS three times and treated with 0.5 ml PI/RNase Staining Buffer (Thermo Fisher Scientific) in the dark for 30 min at room temperature. DNA content was determined immediately by flow cytometry (BD Biosciences). The software FlowJo 7.6 (Ashland, OR, USA) was used to analyze results.

### TUNEL staining

Deparaffinized tissue sections were stained using the APO-BrdU™ TUNEL Assay Kit (A23210; Thermo Fisher Scientific), according to the manufacturer’s instructions.

### Animal studies

The effects of GDF10 overexpression on the tumorigenicity of TNBC cells was examined in 15 female BALB/nude mice (aged 4-6 weeks). Mice were purchased from Shanghai Laboratory Animal Center (SLAC; Shanghai, China) and housed within a dedicated SPF facility with alternating 12 h periods of light and darkness, a constant temperature of 18–23°C, and 55–65% humidity. Animals were acclimatized for about 7 days before inoculation with tumor cells. Aliquots of BT-20 cells (5 x 10^6^ cells in 100 μL of PBS) were injected subcutaneously into the right armpit area of the mice. After that, they were randomly divided into three groups (5 per group): Blank (injected with vehicle only), NC (negative control; injected with cells transfected with a non-coding DNA sequence), and OE (inoculated with GDF10-overexpressing cells). Tumor volume ((length × width 2)/2) was measured with Vernier calipers weekly for four weeks until mice were sacrificed under anesthesia on day 29. Each tumor was excised and weighted, and fragments were wax-embedded for apoptosis determination by TUNEL staining. All animal experiments were performed in accordance with institutional guidelines, following a protocol approved by the Ethics Committees of the Affiliated Hospital of Guizhou Medical University (Guizhou, China).

### Statistical analysis

Data from at least three independent experiments were expressed as the mean ± standard deviation (SD). Student’s t-tests were used for comparisons between two groups. Comparisons among multiple groups were made with one-way analysis of variance (ANOVA) followed by Dunnett’s test. P < 0.05 or P < 0.01 indicated statistically significant differences.

## SUPPLEMENTARY MATERIAL

Supplementary Figures
