# Supplementary material for: GDF10 inhibits proliferation and epithelial-mesenchymal transition in triple‐negative breast cancer via upregulation of Smad7
Source: Aging (Albany NY). 2019 May 31;11(10):3298–314. doi: 10.18632/aging.101983 (PMC6555447; doi:10.18632/aging.101983)
Supplement: Supplementary Figures [file aging-11-101983-s001.pdf]

SUPPLEMENTARY MATERIAL

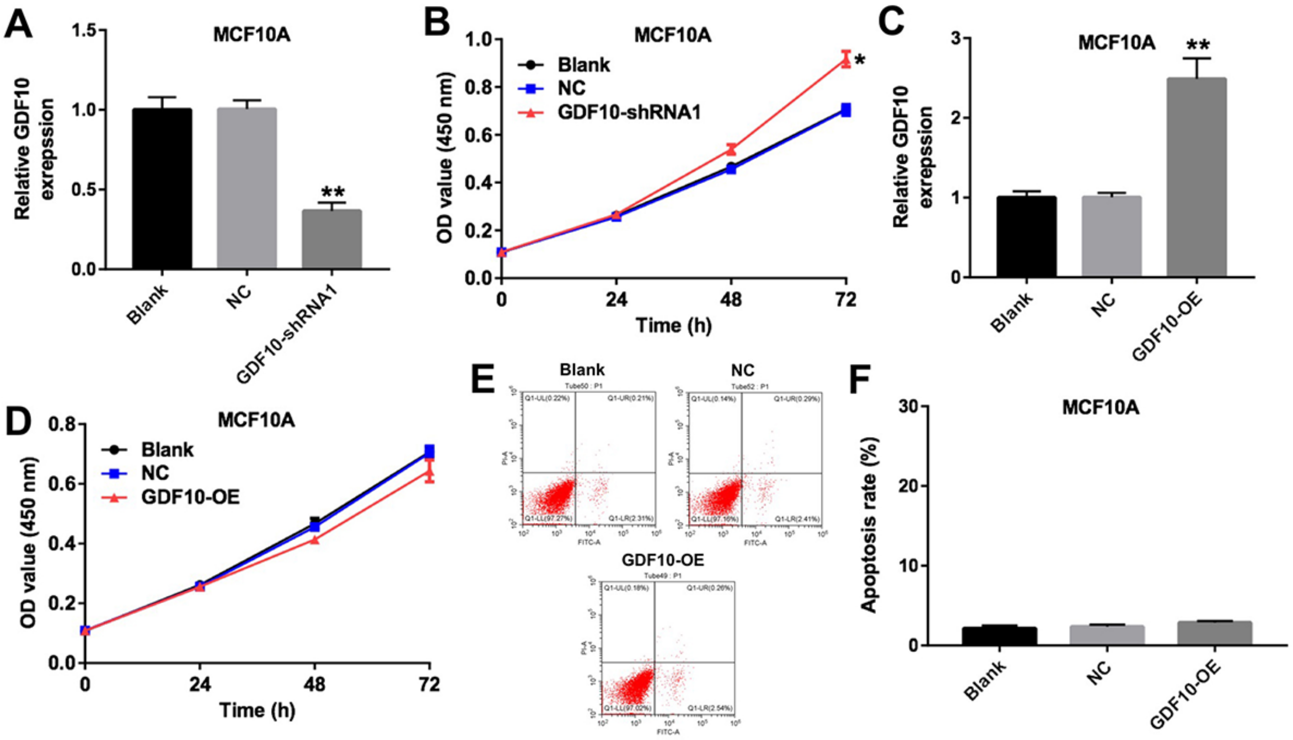

Supplementary Figure 1. Downregulation of GDF10 promotes proliferation of MCF10A cells. (**A**) GDF10 expression at the mRNA levels in MCF10A cells 72 h after transfection with NC or GDF10-shRNA1 for 72 h. **P < 0.01, compared with the NC group. (**B**) Cell proliferation assay results. MCF10A cells were transfected with NC or GDF10-shRNA1 and the CCK-8 assay was conducted at 0, 24, 48, and 72 h. **P < 0.01, compared with the NC group. (**C**) GDF10 expression at the mRNA levels in MCF10A cells 72 h after transfection with NC or GDF10 for 72 h. **P < 0.01, compared with the NC group. (**D**) Cell proliferation assay results. MCF10A cells were transfected with NC or GDF10 and the CCK-8 assay was conducted at 0, 24, 48, and 72 h. **P < 0.01, compared with the NC group. (**E, F**) Apoptosis rates detected through Annexin V/PI double staining and flow cytometry in NC and GDF10-overexpressing MCF10A cells. **P < 0.01, compared with the NC group.

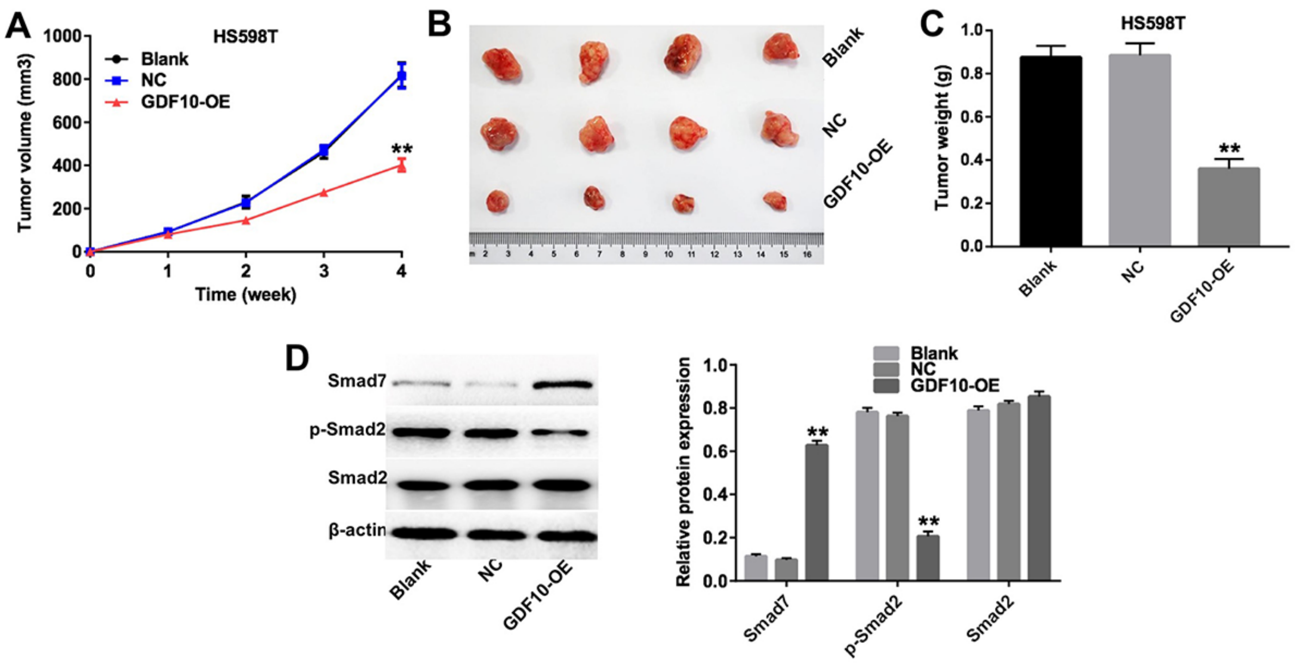

Supplementary Figure 2. GDF10 expression inhibits HS598T xenograft growth. (**A**) Tumor volumes were measured weekly post-inoculation of HS598T cells infected with lentiviruses carrying the GDF10 gene or non-coding controls (NC). **P < 0.01, compared with the NC group. (**B**) Photographs of HS598T xenografts dissected 4 weeks after tumor cell inoculation. (**C**) Tumor weights. **P < 0.01, compared with the NC group. (**D**) The expressions of Smad7, p-Smad2, Smad2 were investigated by western blotting in excised tumor samples. β-actin was used as internal control. Relative protein expression levels were quantified by densitometry and normalized to β-actin. **P < 0.01, compared with the NC group.
